# Association of left ventricular longitudinal strain with central venous oxygen saturation and serum lactate in patients with early severe sepsis and septic shock

**DOI:** 10.1186/s13054-015-1014-6

**Published:** 2015-08-31

**Authors:** Michael J. Lanspa, Joel E. Pittman, Eliotte L. Hirshberg, Emily L. Wilson, Troy Olsen, Samuel M. Brown, Colin K. Grissom

**Affiliations:** Critical Care Echocardiography Service, Intermountain Medical Center, Salt Lake City, UT USA; Division of Pulmonary and Critical Care Medicine, University of Utah, Salt Lake City, UT USA; Department of Pediatrics, University of Utah, Salt Lake City, UT USA

## Abstract

**Introduction:**

In septic shock, assessment of cardiac function often relies on invasive central venous oxygen saturation (ScvO_2_). Ventricular strain is a non-invasive method of assessing ventricular wall deformation and may be a sensitive marker of heart function. We hypothesized that it may have a relationship with ScvO_2_ and lactate.

**Methods:**

We prospectively performed transthoracic echocardiography in patients with severe sepsis or septic shock and measured (1) left ventricular longitudinal strain from a four-chamber view and (2) ScvO_2_. We excluded patients for whom image quality was inadequate or for whom ScvO_2_ values were unobtainable. We determined the association between strain and ScvO_2_ with logistic and linear regression, using covariates of mean arterial pressure, central venous pressure, and vasopressor dose. We determined the association between strain and lactate. We considered strain greater than −17 % as abnormal and strain greater than −10 % as severely abnormal.

**Results:**

We studied 89 patients, 68 of whom had interpretable images. Of these patients, 42 had measurable ScvO_2_. Sixty percent of patients had abnormal strain, and 16 % had severely abnormal strain. Strain is associated with low ScvO_2_ (linear coefficient −1.05, *p* =0.006; odds ratio 1.23 for ScvO_2_ <60 %, *p* =0.016). Patients with severely abnormal strain had significantly lower ScvO_2_ (56.1 % vs. 67.5 %, *p* <0.01) and higher lactate (2.7 vs. 1.9 mmol/dl, *p* =0.04) than those who did not. Strain was significantly different between patients, based on a threshold ScvO_2_ of 60 % (−13.7 % vs. -17.2 %, *p* =0.01) but not at 70 % (−15.0 % vs. −18.2 %, *p* =0.08).

**Conclusions:**

Left ventricular strain is associated with low ScvO_2_ and hyperlactatemia. It may be a non-invasive surrogate for adequacy of oxygen delivery during early severe sepsis or septic shock.

## Introduction

Severe sepsis and septic shock comprise a common and often lethal syndrome that occurs when overwhelming infection results in hypotension and multiorgan failure. Aside from treating the infection, the aim of therapies for severe sepsis and septic shock is to improve a patient’s hemodynamic function with administration of intravenous fluid and vasoactive medications. However, clinicians often are uncertain when to administer these therapies or how much to administer. At least one-third of patients are known to have cardiac insufficiency during severe sepsis or septic shock, which may manifest as an imbalance between oxygen delivery (DO_2_) and oxygen consumption (VO_2_) and can result in low central venous oxygen saturation (ScvO_2_) and elevated lactate [1, 2]. Clinicians often rely on central venous pressure (CVP) and ScvO_2_ to guide therapy aimed at improving DO_2_ [3, 4]. These parameters require the presence of a central venous catheter, which is associated with increased risk of complications, including infection [5]. Aside from the increased risk of catheter placement, there is increasing evidence demonstrating questionable utility of CVP and ScvO_2_ to guide therapy [6–8]. In contrast to catheter measurements of CVP or ScvO_2_, echocardiography may be an attractive means of assessing the adequacy of DO_2_ in patients with septic shock, as it is non-invasive.

Echocardiography is increasingly applied in the critical care environment, as certain echocardiographic parameters predict response to volume expansion [9, 10] while characterizing cardiac systolic and diastolic function. Traditionally, clinicians rely on left ventricular ejection fraction (EF) to assess left ventricular function. However, EF varies with loading conditions and heart rate, and it is poorly reproducible for different observers [11]. Although EF is commonly used as a measure of cardiac systolic function, myocardial strain likely provides a more accurate representation of intrinsic cardiac systolic function. Myocardial strain is an echocardiographic index that has the potential to overcome some of the aforementioned limitations. *Longitudinal strain* is defined as the percent change of length of an object. When applied to the ventricle, longitudinal strain is simply the percentage distance that the endocardial wall shortens along its length. Strain appears to have value in detection of early changes of myocardial ischemia [12]. Although strain is not a commonly acquired measurement in bedside echocardiography, it is possible to do real-time quantitation of strain at the bedside [13].

We sought to determine primarily whether left ventricular longitudinal strain is associated with adequate DO_2_, either by reduced ScvO_2_ or by hyperlactatemia, and secondarily to compare strain with EF in patients with severe sepsis and septic shock.

## Methods

### Study design

This prospective, observational study was conducted between September 2008 and April 2010 at the Intermountain Medical Center, an academic tertiary care hospital in Murray, UT, USA. Patients admitted to the 24-bed shock trauma intensive care unit (ICU) or the 12-bed respiratory ICU were eligible for inclusion. The Intermountain Medical Center Institutional Review Board (number 1009957) approved this study. All patients or their legally authorized representatives provided written informed consent.

### Patients

Study investigators prospectively screened patients admitted to study ICUs with severe sepsis or septic shock as defined by the American College of Chest Physicians/Society of Critical Care Medicine consensus criteria [14]. We included patients who had the following characteristics: (1) at least 14 years of age, (2) a suspected infection, (3) two or more systemic inflammatory response syndrome criteria, and (4) either severe sepsis (end-organ dysfunction) or septic shock (systolic blood pressure > 90 mmHg after an intravenous fluid challenge of ≥20 ml/kg with evidence of organ dysfunction or serum lactate ≥4 mmol/dl). We excluded patients with a primary diagnosis of acute coronary syndrome or major cardiac dysrhythmia, presence of pericardial tamponade, presence of mitral stenosis, known diagnosis of severe pulmonary hypertension, lack of sinus rhythm during echocardiography, or a contraindication to central venous catheterization, as well as patients for whom the family or clinician declined to pursue intensive therapy.

Patients were treated according to the Surviving Sepsis Campaign guidelines [4]. Specifically, in patients requiring a central venous catheter, treatment followed an early goal-directed therapy protocol targeting a mean arterial pressure (MAP) of ≥65 mmHg, CVP ≥8 mmHg, and ScvO_2_ ≥70 % [3].

### Transthoracic echocardiography

We obtained transthoracic echocardiograms (TTEs) using either a Philips iE33 or CX50 system (Philips Medical Systems, Bothell, WA, USA). We obtained the TTEs within the first 6 hours of determining that the patient met the inclusion criteria. All TTEs were interpreted by the first author (MJL), who is a level 3 echocardiographer and a testamur of the National Board of Echocardiography. We assessed intraobserver reliability by having the interpreter do a blinded repeat read several months later and calculated an intraclass correlation coefficient (ICC =0.8). We used the Image-Arena platform (TomTec Imaging Systems, Unterschleissheim, Germany) to perform speckle tracking for left ventricular longitudinal strain. We selected standard apical four-chamber views for strain analysis. We selected the best available single cardiac cycle with regard to image quality and measured longitudinal strain of the endocardium. We rejected images due to poor image quality if we could not track two or more adjacent segments in the apical four-chamber view. We defined abnormal strain as greater than −17 % and severely abnormal strain as greater than −10 % (higher numbers are worse) in accordance with previously published literature describing patients with septic shock [15]. We defined hyperdynamic strain as less than −22.1 % [16]. We measured EF using the monoplane Simpson’s method of disks from the apical four-chamber view and defined severely abnormal EF as <30 % and hyperdynamic EF as > 70 % [17]. We calculated cardiac output using velocity–time integration derived from pulse wave Doppler imaging at the left ventricular outflow tract. All echocardiographic interpretations were blinded to all clinical data at the time of image analysis.

### Clinical data

In addition to analysis by age and sex, we assessed disease severity by calculating admission Acute Physiology and Chronic Health Evaluation II score [18]. We assessed comorbidities using the Elixhauser comorbidity score [19]. We recorded the etiology of sepsis according to predefined categories of thoracic, abdominal, skin or soft tissue, central nervous system, urinary, central venous catheter, or endocarditis. We calculated Sequential Organ Failure Assessment (SOFA) scores according to a previously published methodology [20]. We recorded CVP, MAP, and ScvO_2_ at the time of echocardiography in patients who had a catheter capable of continuous ScvO_2_ monitoring (PreSep; Edwards Lifesciences, Irvine, CA, USA). As part of the screening process, all patients had serum lactate recorded at the time of their enrollment in the study. We also measured vasopressor infusions at the time of echocardiography, expressed by summing administered vasopressors as a norepinephrine-equivalent dose: norepinephrine + epinephrine + (0.01) dopamine + (5) vasopressin + (0.45) phenylephrine, where vasopressin dose is expressed in units per minute and all other vasopressors are expressed in micrograms per kilogram per minute [21].

### Statistical analysis

We performed linear regression between strain and ScvO_2_ using covariates of age, SOFA score, MAP, CVP, and vasopressor dose to evaluate the fundamental relationship between strain and ScvO_2_. We selected our covariates on the basis of our clinical expectation that they would be related to ScvO_2_ (or lactate) and cardiac function. As a prespecified analysis, we then performed logistic regression to evaluate relevant clinical thresholds by using the same covariates to predict low ScvO_2_, defined as <70 %. In an exploratory analysis, we repeated the logistic regression model using a ScvO_2_ threshold of <60 %. As a prespecified analysis, we repeated these regression models substituting left ventricular EF for strain. EF was excluded from the original models owing to high collinearity between strain and EF. We compared central tendencies with the Mann–Whitney *U* test. We calculated non-parametric receiver operating characteristic curves for thresholds of 70 % and 60 % and determined areas under the curve according to the method of Hosmer and Lemeshow [22]. As a prespecified analysis, we compared central tendencies of lactate with patients who had abnormal versus normal strain and abnormal versus normal EF, and we performed linear regression, as above, substituting lactate for ScvO_2_. As a prespecified analysis, we performed logistic regression, as above, substituting hyperlactatemia (lactate ≥4 mmol/dl) for the dependent variable. In further exploratory analyses, we performed similar regression models for ScvO_2_ and lactate, substituting cardiac output instead of strain and retaining the same covariates of MAP, CVP, and vasopressor dose for the initial models. In all regression analyses, we employed stepwise backward elimination using an exclusion threshold of *p* >0.20 and change in estimate <10 %. All analyses were performed with Stata v12 software (StataCorp, College Station, TX, USA).

## Results

We performed transthoracic echocardiography in 89 patients with severe sepsis or septic shock within the first 6 hours of presentation to the emergency department. We were able to measure strain in 68 patients and ScvO_2_ in 52 patients. We were able to measure both strain and ScvO_2_ in 42 patients. Basic demographic and disease severity data are characterized in Table 1. At the time of echocardiography, about one-third of the patients were undergoing mechanical ventilation and about one-half of the patients were receiving an intravenous vasopressor infusion. Thirty-day all-cause mortality was 16.9 %.

**Table 1:** Characteristics of all enrolled patients and patients with measurable central venous oxygen saturation and strain

	Severe sepsis and septic shock, N =89	Subset with measurable ScvO_2_ and strain, N =42
Females (%)	48.3	52.4
Age, yr	57 (45, 65)	57 (41, 66)
APACHE II score	25 (20, 32)	27 (21, 34)
Elixhauser comorbidity score	5 (3, 6)	5 (3, 6)
Source of sepsis (%)		
Thoracic	39.3	45.2
Abdominal	13.5	16.7
Skin/soft tissue	13.5	9.5
Central nervous system	1.1	2.4
Urinary	18.0	19.0
Central venous catheter	6.7	4.8
Endocarditis	1.1	0
On mechanical ventilation (%)	31.8	35.7
Mean arterial pressure (mmHg)	70 (65, 80)	71 (65, 79)
Fluid administered before TTE (L)	3.1 (1.2, 5.4)	3.0 (1.0, 5.0)
Receiving vasopressor (%)	39.3 %	50.0 %
Norepinephrine-equivalent vasopressor dose among patients with shock (μg/kg/min)	0.1 (0.05, 0.2)	0.1 (0.05, 0.15)
With indwelling central venous catheter (%)	60.7 %	100 %
Central venous pressure (mmHg)	11 (8, 14)	11 (10, 14)
Longitudinal strain (%)	−15.3 (−18.9, −12.1)	−16.3 (−19.5, −12.3)
Ejection fraction (%)	60.5 (44.2, 70.0)	61.1 (44.0, 70.0)
Stroke volume (ml)	61.2 (44.8, 77.3)	60.6 (43.6, 76.4)
Cardiac output (L/min)	6.2 (4.5, 7.9)	6.1 (4.5, 7.4)
Hemoglobin (g/dl)	10.9 (9.6, 12.7)	11.4 (9.7, 12.9)
SpO_2_ (%)	97 (94, 99)	97 (95, 99)
ScvO_2_ (%)	66 (57, 75)	66 (57, 73)
Serum lactate (mmol/dl)	2.1 (1.35, 3.3)	2.1 (1.3, 3.3)

*APACHE II* Acute Physiology and Chronic Health Evaluation II, *ScvO*_*2*_ central venous oxygen saturation, *SpO*_*2*_ peripheral capillary oxygen saturation, *TTE* transthoracic echocardiogram

Medians and interquartile ranges are reported for continuous data

**Fig. 1:**
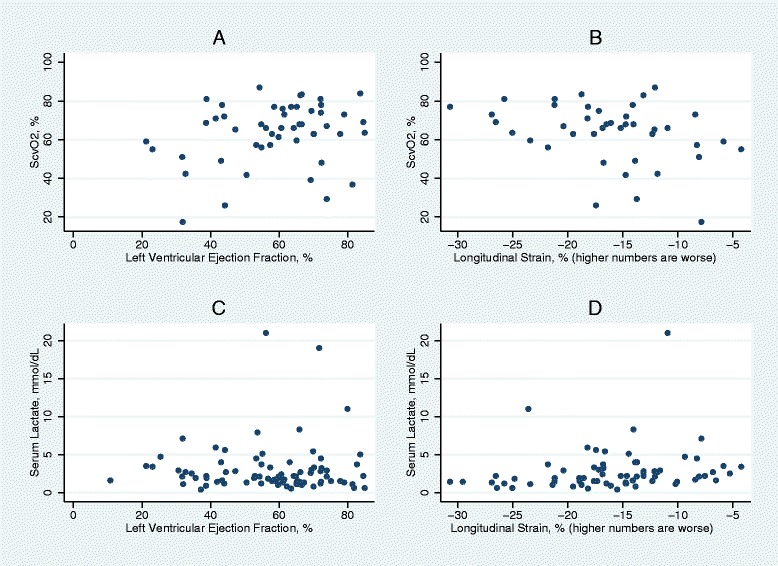
Scatterplots of (**a**) left ventricular ejection fraction (EF) and central venous oxygen saturation (ScvO_2_), (**b**) longitudinal strain and ScvO_2_, (**c**) EF and lactate, and (**d**) strain and lactate. These graphs illustrate that elevated lactate and low ScvO_2_ may be observed with elevated EF but are less often observed with normal strain

Abnormal strain (greater than −17 %) was present in 60 % of patients, and severely abnormal strain (greater than −10 %) was present in 16 % of patients. The linear and logistic regression models demonstrate that strain is associated with low ScvO_2_ (Table 2). Patients with severely abnormal strain had significantly lower ScvO_2_ than those with normal or mildly reduced strain (56.1 % vs. 67.5 %, *p* <0.01). Although longitudinal strain was significantly different between patients with ScvO_2_ <60 % and those with ScvO_2_ ≥60 % (−13.7 % vs. −17.2 %, *p* =0.01), this trend did not maintain statistical significance in the primary analysis with a threshold of 70 % (−15.0 % vs. −18.2 %, *p* =0.08). The scatterplot of ScvO_2_ and strain is depicted in Fig. 1. We observed that 72.3 % of patients had normal EF. Left ventricular EF also demonstrated a relationship with ScvO_2_ in linear regression (ICC 0.29, 95 % confidence interval [CI] 0.02–0.057, *p* =0.036) and in logistic regression for ScvO_2_ <60 % (odds ratio [OR] 0.92, 95 % CI 0.86–0.97, *p* =0.004). EF was not significantly associated with ScvO_2_ <70 % (OR 0.98, 95 % CI 0.95–1.02, *p* =0.352).

**Table 2:** Linear and logistic regression models for low ScvO_2_

Linear regression for ScvO_2_	Coefficient	95 % Confidence interval	*p* Value
Longitudinal strain	−1.05	−1.78, −0.32	0.006
Central venous pressure	−0.95	−1.85, −0.04	0.041
Logistic regression ScvO_2_ <60 %	Odds ratio	95 % Confidence interval	*p* Value
Longitudinal strain	1.23	1.04, 1.45	0.016
Central venous pressure	1.26	1.02, 1.55	0.030
Logistic regression ScvO_2_ <70 %	Odds ratio	95 % Confidence interval	*p* Value
Longitudinal strain	1.11	0.99, 1.26	0.079

*ScvO*_*2*_ central venous oxygen saturation

Initial model included covariates of age, Sequential Organ Failure Assessment score, central venous pressure, mean arterial pressure, and norepinephrine-equivalent vasopressor dose. Linear regression *R* ^2^ =0.27. Areas under the curve for the logistic regression models were 0.84 for ScvO_2_ <60 % and 0.75 for ScvO_2_ <70 %

We observed no difference in the area under the receiver operating characteristic curve (AUCROC) between strain and EF for thresholds of ScvO_2_ <70 % (0.67 vs. 0.57, *p* =0.22) or ScvO_2_ <60 % (0.74 vs. 0.70, *p* =0.83).

We noted an association with strain and lactate as well. Among the 68 patients in whom we were able to measure strain, patients with abnormal strain had higher serum lactate values (median 2.4 mmol/dl vs. 1.5 mmol/dl, *p* =0.03). We also noted higher lactate values in patients with severely abnormal strain (2.7 mmol/dl vs. 1.9 mmol/dl, *p* =0.04), an association that remained in the subset of 42 patients with measurable strain and ScvO_2_ (3.45 mmol/dl vs. 2.00 mmol/dl, *p* =0.05). We did not detect an association between strain and lactate in linear regression (ICC 0.09, *p* =0.13) or logistic regression (OR 1.07, *p* =0.23). We observed no differences in lactate among patients with abnormal EF (2.5 mmol/dl vs. 2.1 mmol/dl, *p* =0.33) or severely abnormal EF (2.8 mmol/dl vs. 2.0 mmol/dl, *p* =0.13). We did not detect an association between EF and lactate in linear regression (ICC 0.00, *p* =0.86) or logistic regression (OR 1.00, *p* =0.86). We found no associations between strain, EF, and 30-day all-cause mortality.

In an exploratory analysis, we examined the association between echocardiographically measured stroke volume and ScvO_2_ and lactate using regression analysis. Stroke volume (measured in milliliters) was associated with ScvO_2_ (ICC 0.21, *p* =0.030) but not with lactate (*p* =0.20). In logistic regression, stroke volume was associated with ScvO_2_ <60 % (OR 0.0.93, *p* =0.016, AUCROC 0.80) but not with ScvO_2_ <70 % (OR 0.98, *p* =0.33, AUCROC 0.58).

In post hoc analyses, we examined the potential additional value of strain in patients with normal EF. Of patients with normal EF and measurable strain (n =46), 50 % had abnormal strain and 6.5 % had severely abnormal strain. Among patients with normal EF, lactate was higher in patients with abnormal strain (median 2.2 mmol/dl vs 1.4 mmol/dl, *p* =0.01). We observed no difference in regard to ScvO_2_ (66 % vs. 69 %, *p* =0.29) in this group, nor did we observe a difference in strain between patients with a normal EF and ScvO_2_ <60 % and those with normal EF and ScvO_2_ ≥70 % (−16.7 % vs. −17.3 %, *p* =0.38).

## Discussion

Impaired left ventricular longitudinal strain correlates with reduced ScvO_2_ and elevated serum lactate in patients with early severe sepsis or septic shock. The value of this study lies in the demonstration of an association between a non-invasive index of cardiac function and a more traditional method of assessing the adequacy of DO_2_ for a given ScvO_2_. The current trend in ICU care is to use non-invasive hemodynamic monitoring [23]. Validation, including testing of protocols that explicitly incorporate strain measurements, would be required before broad application of strain in clinical practice for management of patients with septic shock. Nonetheless, our study suggests that strain may have some value in future research as a non-invasive marker of cardiac function in septic shock.

Strain may have some applications in outpatient cardiology practice, but few studies have been conducted in the ICU. Strain appears to be more sensitive than EF for detecting cardiac dysfunction in sepsis [15, 24], although there is poor correlation between strain rate and troponin elevation in sepsis [25]. Our study is the first to include an evaluation of the correlation of strain and ScvO_2_ in patients with severe sepsis and septic shock. Our study also has the advantages of prospective enrollment and early measurement, as all patients underwent imaging within 6 hours of meeting criteria for severe sepsis.

Because EF is dependent on cardiac loading and heart rate, a patient with tachycardia and preload deficit may have a hyperdynamic EF (>70 %) and still have inadequate cardiac output. We found that though EF generally correlates with ScvO_2_, we still observed some patients with hyperdynamic EF and ScvO_2_ <60 % (n =4). Among those four patients who had measurable strain, strain was abnormal (n =2). Our study was insufficiently powered to determine whether strain was better than EF at predicting ScvO_2_ <60 %, and we did not directly compare strain and EF in our regression models, owing to their high collinearity. Elevated serum lactate, another biomarker of inadequate cardiac output, appeared to have no association with EF, with the highest observed lactate levels occurring in patients with normal or hyperdynamic EF. Excepting one outlier, we observed high lactate levels in patients with abnormal strain. We cannot exclude the possibility that lactate elevation resulted in part from increased muscle production in patients with preserved EF who were receiving catecholamine infusions.

We selected ScvO_2_ as an outcome of interest because it is a commonly applied method for assessing the adequacy of DO_2_ during sepsis, where cardiac function plays an inextricable role. Aside from technical problems with measuring ScvO_2_, such as equipment calibration or location of the distal tip of the catheter, the ScvO_2_ value may have theoretical limitations. Unlike mixed venous oxygen saturation (SvO_2_), ScvO_2_ ignores the contribution of deoxygenated blood from the coronary sinus [26]. As global measures of oxygenation, ScvO_2_ and SvO_2_ are unable to measure regional hypoperfusion. Sepsis can also affect hemoglobin’s affinity for oxygen [27] or result in a state of decreased oxygen extraction and microvascular shunting [28, 29]. Where such shunting is present, patients may have an artificially high ScvO_2_ despite substantially impaired DO_2_. In such cases, a patient could have hypoperfusion of vascular beds with normal or high SvO_2_ or ScvO_2_. We had initially hoped that strain would identify patients with microvascular shunting and otherwise adequate preload and normal EF. However, our data are unable to support such an inference. We observed only 16 patients with CVP ≥8 mmHg, EF ≥50 %, and ScvO_2_ ≤70 %, and half of these patients had normal strain (median −16.7 %; interquartile range −20.3 %, −14.0 %).

Some investigators have proposed lactate as an alternative for assessing the adequacy of cardiac output, as it should be elevated in the setting of decreased DO_2_, decreased oxygen extraction, or microvascular shunting [30]. A limitation of lactate in our study is that only 18 % of our patients had lactate elevation >4 mmol/dl, suggesting it may be a relatively insensitive biomarker compared with ScvO_2_, which was <70 % in 63 % of patients. Another limitation of lactate in our study is that the lactate was measured at the time of screening and enrollment, not at the time of echocardiography. Thus, whereas the ScvO_2_ was obtained simultaneously with the echocardiogram, the lactate measurement was not.

We initially chose a threshold of ScvO_2_ <70 % as abnormal. We selected this threshold because it is commonly used as trigger for blood transfusion or inotropic support in septic shock [3, 4]. However, we found in secondary analysis that the association between strain and ScvO_2_ was better when we used a threshold ScvO_2_ of <60 %. The literature appears to support the finding that 60 % may be a more clinically relevant threshold for assessing cardiac function. Among patients with acute decompensated heart failure who are receiving inotropes, an optimal threshold ScvO_2_ of 60 % was determined in sensitivity analyses to correlate with adverse outcomes [31]. A threshold ScvO_2_ of 60 % also appears to discriminate increased mortality in ICU patients [32].

We were able to measure strain in 76 % of patients and EF in 93 %. Image quality was the sole reason for failure to measure strain. The proportion of patients with measurable EF versus measurable strain is partly misleading because traditional methods of measuring EF allow for interpreter subjectivity in poor-quality images, where the observer may “fill in” the missing segments. We analyzed the same image to calculate both EF and strain. Strain, which uses a computer algorithm to track image speckles, cannot overcome poor-quality images with current technology. Many of the patients in the ICU have poor-quality images owing to body habitus, positioning, and mechanical ventilation. Furthermore, longitudinal strain is not immune to changes in either preload or afterload [33]. We attempted to account for this in our regression models by including CVP and vasopressor dose as covariates, although these are imperfect correlates of preload and afterload. None of our patients had significant aortic stenosis, which could affect strain. Similarly, very few patients in our study were hypovolemic at the time of the study, as CVP was ≥8 mmHg in 75 % of patients. Even at this relatively early stage of sepsis, most patients had received 2–4 L of crystalloid before undergoing echocardiography. Our findings may not be generalizable to a septic patient with severe hypovolemia.

Several criticisms exist regarding the reproducibility of strain. Values may be specific to the vendor’s algorithm and may not necessarily translate from one software package to another [34]. Other authors have posited that three-dimensional strain may improve the reproducibility of measurements as movement of the heart in and out of the imaging plane is ignored in two-dimensional strain analysis [35]. In our own study, our intraobserver reliability was good (ICC 0.80), but interobserver reliability may not be as good. Although not explored in this study, there are other echocardiographic measurements of contractility in the longitudinal dimension that may be easier to image, such as mitral annular plane systolic excursion (MAPSE) and mitral annulus systolic velocity (*S*′) measured by tissue Doppler imaging. Although strain by speckle tracking is technically more difficult to image than MAPSE or *S*′, it avoids the problems that occur when the image is off axis and the mitral annulus movement is not aligned with the probe.

We selected longitudinal strain a priori for our analysis and compared it with EF. There exist several other echocardiographic measurements that may also have an association with ScvO_2_ or lactate. In our exploratory analysis, we observed that echocardiographically derived cardiac output, which is typically easier to measure than strain, has an excellent association with ScvO_2_. Most strain analysis packages generate a large amount of data, including strain rate, velocity, displacement, and time to peak for all the aforementioned variables, as well as the affording the ability to assess every segment. Given the small numbers, we were wary of the problems of multiple comparisons and therefore did not want to investigate too many variables out of concern of creating a type I error.

Perhaps of greatest clinical interest is whether strain will perform better than EF to identify potential left ventricular systolic dysfunction in patients with reduced ScvO_2_ or elevated lactate. Unfortunately, we are unable to answer this question based on the present study, owing to insufficient power to detect a difference. With recent publications suggesting treatment of severe sepsis and septic shock without central venous catheters [6, 7] and a cultural transition away from invasive monitoring, there may be fewer opportunities to observe ScvO_2_ measurements in future studies of severe sepsis and septic shock.

This study has several strengths, including its prospective enrollment of patients, the use of a measure that is less susceptible to interpreter bias than traditional EF, and the early acquisition of images within the first 6 hours of meeting criteria for severe sepsis or septic shock. Although normal values for strain are yet to be determined, our chosen cutoff value for severely abnormal strain was based on a previously published study [15] in patients with sepsis rather than derived ad hoc from the present study data, which increases the likelihood of generalizability. Limitations of this study include sample size and performance at a single center. Strain was measurable in only 76 % of patients owing to image quality. We analyzed a single cardiac cycle rather than an average of several consecutive cycles. We did not compare longitudinal strain and biplane EF. Rather, our strain and EF analyses were restricted to single-plane analysis (four-chamber view), which may miss potential information visible in other echocardiographic views. Strain analysis requires specialized software typically not included in most critical care echocardiography systems, which currently limits its generalizability and applicability. Our chosen outcome, ScvO_2_, may be a poor representation of the adequacy of DO_2_ in the critically ill patient being treated with vasopressors. As this study required informed consent from either a critically ill patient or the patient’s surrogate, there is a likelihood of selection bias, as consent is not always obtainable from critically ill patients. Fewer than half of the enrolled patients actually had measureable strain and an ScvO_2_ measurement, and those patients were more likely to be on vasopressors, which raises concerns of generalizability. Our study cohort included patients who had severe sepsis rather than septic shock, with an associated overall lower severity of illness. These findings may not necessarily be generalizable to all patients with severe sepsis or septic shock.

## Conclusions

We found an association between longitudinal strain of the left ventricle and ScvO_2_ and serum lactate in patients with early severe sepsis and septic shock. Measurement of strain may be useful in future research in septic shock as a non-invasive means of assessing cardiac function.

## Key messages

- Longitudinal strain, a relatively novel measurement of ventricular function, is frequently abnormal in patients with septic shock.
- Worsened longitudinal strain is associated with lower ScvO_2_ and higher lactate levels, suggesting inadequate DO_2_.
- Although we observed a relationship between EF and ScvO_2_, we did not observe a similar relationship with EF or lactate.

## Abbreviations

APACHE II: Acute Physiology and Chronic Health Evaluation II
AUCROC: Area under the receiver operating characteristic curve
CI: Confidence interval
CVP: Central venous pressure
DO_2_: Oxygen delivery
EF: Ejection fraction
ICC: Intraclass correlation coefficient
ICU: Intensive care unit
MAP: Mean arterial pressure
MAPSE: Mitral annular plane systolic excursion
OR: Odds ratio
*S*′: Mitral annulus systolic velocity
ScvO_2_: Central venous oxygen saturation
SOFA: Sequential Organ Failure Assessment
SpO_2_: Peripheral capillary oxygen saturation
SvO_2_: Mixed venous oxygen saturation
TTE: Transthoracic echocardiogram
VO_2_: Oxygen consumption
